# Opportunistic Screening of Oral Potentially Malignant Disorders: A Public Health Need for India

**DOI:** 10.1200/JGO.19.00350

**Published:** 2020-05-01

**Authors:** Priya Mohan, Ann Richardson, John D. Potter, Patricia Coope, Margaret Paterson

**Affiliations:** ^1^ School of Health Sciences, University of Canterbury, Christchurch, New Zealand; ^2^ Centre for Public Health Research, Massey University, Wellington, New Zealand; ^3^ Library, University of Canterbury, Christchurch, New Zealand

## Abstract

**PURPOSE:**

Oral cancer (OC) is the leading cancer in 25% of Indian cancer registries, and 80% of OCs are diagnosed in advanced stages. OC screening is a topic of debate. Studies from other countries have used a variety of study designs as OC screening strategies. There are not many studies from India on strategic screening, and there is a need to review the literature to provide insights and knowledge about screening programs. The purpose of this narrative review is to present broad epidemiologic evidence on the OC burden in India, to discuss and summarize the currently available evidence for OC screening strategies, and to highlight a feasible opportunistic screening strategy for addressing OC burden in India.

**METHODS:**

Medline and EMBASE were used to identify articles. Data from GLOBOCAN and government reports were obtained from websites. As many key concepts and divergent views cannot be addressed with a single research question, a narrative review was considered appropriate, but to ensure a comprehensive literature search, a systematic review search strategy was used.

**RESULTS:**

OC rates are rising more rapidly in India than projected. Wide variations in OC incidence within India reflect regional diversity of risk factors. Studies abroad have demonstrated the feasibility of opportunistic screening of oral potentially malignant disorders by dentists; however, although recommendations exist in India, no studies of opportunistic screening by dentists have been reported.

**CONCLUSION:**

The projected major increases in the OC burden necessitate an OC screening program; opportunistic screening of high-risk groups by dentists using oral visual examination is recommended as a cost-effective strategy. As a way forward, a pilot project to assess the feasibility of regional opportunistic screening is in progress.

## INTRODUCTION

Globally, oral cancer (OC) is the 16th most common cancer.^1^ In Asia, specifically South Central and Southeast Asian countries, the prevalence of OC is high.^1-3^ India has been considered the global epicenter of OC.^4^ In India, it is the most common cancer among men and the third most common cancer among women.^1,5^ It is the leading cancer in 7 of the 27 Indian cancer registries.^6^ OC accounts for 30% of all cancers in India.^7,8^ Indian cancer registries cover < 10% of the population, and most are located in urban areas,^9^ whereas 72% of the population is rural, and some of the most populous states do not have cancer registries.^10^ Furthermore, there are geographic gaps in the availability of cancer diagnostic services because health resources in India are inequitably distributed and vary in their accessibility.^7^ Thus, the reported incidence rates may be lower than the actual incidence rates. Delayed diagnosis is the main cause of the high morbidity/mortality and economic burden of OC.^2^ Oral potentially malignant disorders (OPMDs) are the forerunners of OC.^11,12^ Detection and treatment of OPMDs is an important OC control strategy, and screening can potentially prevent premature death and suffering.^13^ However, because of the lack of evidence on the efficacy of population-based screening and the failure to meet the overall criteria specified for new population-based screening programs, no national screening programs exist for OC in any country.^14^ Some developed countries provide oral screening as part of general health screening. However, for the Indian population, there are no reports of such screening programs. Without such programs, opportunistic screening is the only strategy for the reduction of the OC burden and is a national priority.^15^

**CONTEXT**

**Key Objective**
To summarize the growing burden of oral cancer in India and trends in oral cancer screening programs and to evaluate the most pragmatic approach to a secondary prevention method in India while cognizant of its resource constraints.
**Knowledge Generated**
The summary of evidence from this review will support additional research into opportunistic screening as an oral cancer control strategy in India.
**Relevance**
Oral potentially malignant disorders manifest as distinct lesions before progressing to oral cancer and can be clinically detected by conventional oral examination. Dental colleges provide specialist services for detection and treatment of these disorders. This review describes the burden of oral cancer and presents opportunistic screening in dental colleges as a clinical approach for oral cancer control in India.

This review was undertaken to present broad epidemiologic evidence of the OC burden in India, discuss and summarize the various OC screening strategies, and highlight a feasible screening strategy that may be able to address the OC burden in India. Because there were many key concepts and divergent views about OC screening that could not be addressed with a single research question, a narrative review was considered more appropriate instead of a systematic review or meta-analysis; thus, we undertook this narrative review. To our knowledge, this review is the first to synthesize and summarize the literature from an Indian perspective.

## METHODS

**FIG A1:**
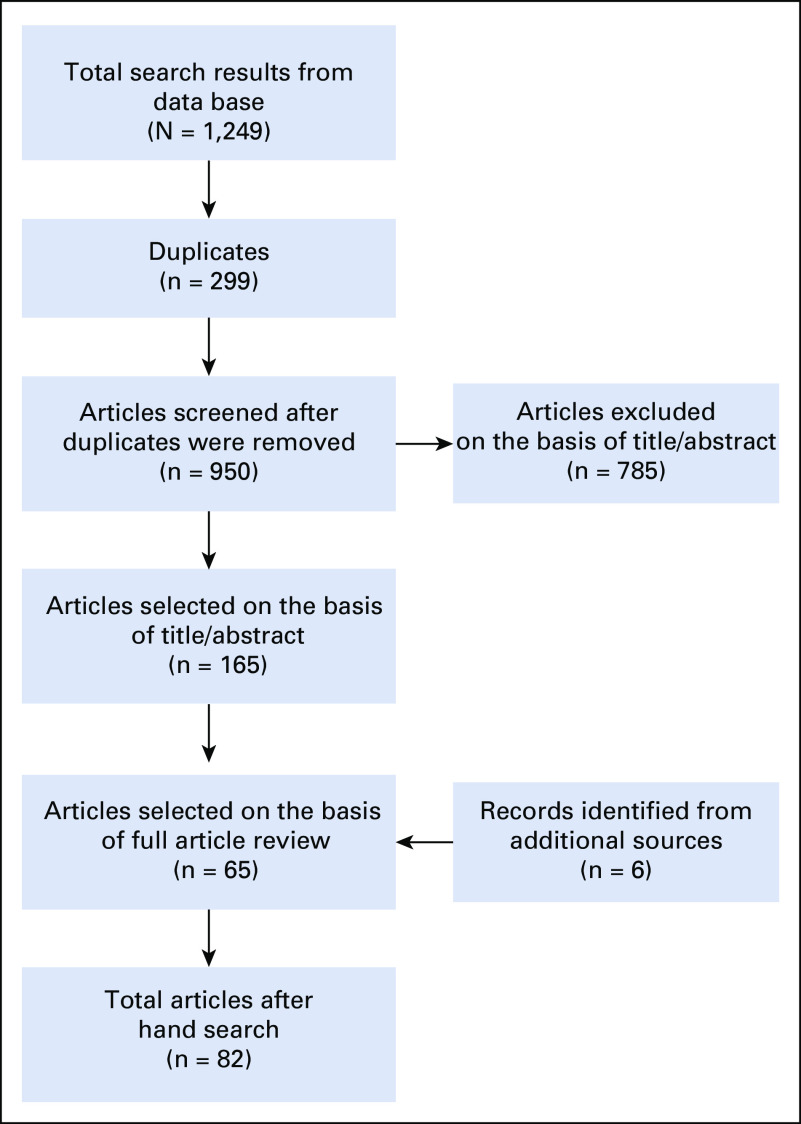
Search strategy.

A structured search of articles on OC and screening from journals published up to September 2019 was undertaken using the EMBASE and Medline databases (Appendix Fig A1). Only articles published in English were included. The main search terms used were oral, cancer, precancer, burden, screening, and India; supplementary key words used were mouth, tongue, and potentially malignant disorders. Additional relevant articles cited in the reference lists of retrieved articles were obtained manually through Google Scholar.

We also reviewed the following websites for updated information: GLOBOCAN 2012 and 2018, National Centre for Disease Informatics and Research, National Program for Cancer Registries, International Institute for Population Studies, International Agency for Research on Cancer, and the Indian Council for Medical Research. These were added to the reference list. Furthermore, after a reviewer’s suggestions on an earlier draft of this article, additional articles were referenced. An expert health sciences librarian assisted in the literature search.

## RESULTS

### Descriptive Epidemiology

#### OC incidence.

The age-standardized incidence of OC varies geographically, and this corresponds to regional differences in the prevalence of risk factors.^7^ According to GLOBOCAN 2018, OC was the 16th most common cancer globally; the number of incident OC cases was 354,864 (246,420 in males; 108,444 in females). The overall age-standardized incidence rate is 4.0 per 100,000 and 5.8 and 2.0 per 100,000 for males and females, respectively.^1^ Although OC is a problem in high-, low-, and middle-income countries, two thirds of OCs are reported from developing countries.^16^ It is more common in southern Asia, especially India and Sri Lanka.^17^ In India, the overall age-standardized incidence rate is 9.1 per 100,000, with sex-specific age-standardized incidence rates of 13.9 and 4.3 per 100,000 among males and females, respectively.^1^ In 2018, India was estimated to have 119,992 incident cases of OC; of these, 92,011 (76.7%) were reported in men.^1^ The number of people diagnosed with OC in India is increasing. This is evidenced by the differences in the number of incident OC cases between GLOBOCAN 2012 and 2018 data. The latter, in fact, surpassed the projection of GLOBOCAN 2012.^18^ In most developed countries, cancer registries cover the national population (or a known proportion), but in developing countries, cancer registries may cover only the populations of major cities.^19^ Moreover, in India, some of the most populous states do not have cancer registries, which makes the actual OC incidence uncertain because of under-reporting.^10^ The age-standardized incidence rates for males and females vary more than five-fold across Indian states.^10^

#### Trends.

Globally, there has been a decrease in the age-standardized incidence of OC,^20^ but comparisons across anatomic locations reveal that tongue cancer incidence has been increasing among the young.^16,20^ In India, there have been differences in the trends in the age-standardized OC incidence rates among cancer registries. For instance, the Mumbai cancer registry showed a reduction in rates for men between 1986 and 2000,^2^ but a study from Ahmedabad between 1980 and 2010 showed an increase in the age-specific incidence of OC, with most of the diagnosed cases being among men < 45 years of age.^12^ There has also been a reduction in the male-to-female ratio, and the Bangalore cancer registry showed an age-standardized incidence rate of OC in women (10.0 per 100,000) that exceeded that of men (6.3 per 100,000).^21^ A study on the time trends of various cancers in India reported that between 1990 and 2016, there was a 6.4% reduction in the age-standardized incidence of OC.^10^

#### Projections.

Crude incidence projections indicate that the number of people with OC will continue to increase.^7^ The demographic profile of the relatively young Indian population is changing rapidly because of increasing population growth and aging.^22^ Both of these trends, as well as increasing life expectancy, increase the overall burden of cancer.^22,23^ Compared with the overall population growth, the elderly population (age ≥ 60 years) is growing three times faster in India,^24^ and because the incidence of OC increases with age,^25,26^ advancing age is a risk factor for OC in India.^25,27^ According to a 3-year Population Based Cancer Registry (PBCR) of India report in 2016, mouth cancer was the leading cancer among males in 7 of 27 PBCRs, and tongue and mouth cancers together are projected to increase from 106,794 cases in 2015 to 144,357 cases in 2020.^6^ This is a much larger increase than that projected by GLOBOCAN 2012 for India, which is from 77,000 cases in 2012 to 102,579 cases by 2020.^18^ The estimated number of incident OC cases is predicted to rise from 119,992 in 2018 to 126,416 in 2020.^1^ This is much higher than the projections of GLOBOCAN 2012 but lower than the prediction of the Indian Council of Medical Research.^6^ It seems likely that GLOBOCAN selectively obtained data from only good-quality PBCRs in India.^28^ What is evident from the literature is that the number of OC cases is increasing faster than the estimated projected numbers, irrespective of the source of those projections. The proportion of the Indian population age ≥ 60 years has been increasing three times faster than the total population growth rate; consequently, the cancer burden is expected to increase, but some cases will go undiagnosed because of poor health literacy and lack of accessibility and affordability of health services.^22^

#### Mortality.

OC accounts for 23% of cancer-related deaths in India,^29^ and among men, OC is the leading cause of cancer death.^17^ According to GLOBOCAN estimates for 2018, there were 177,384 global deaths as a result of OC, with 119,693 (67.5%) in men. India accounted for > 60% of these male deaths (n = 72,616).^1^ There is an increased risk of premature mortality among smokeless tobacco (SLT) users from India.^30^ Relative risks of premature mortality among SLT users compared with non-SLT users varied between 1.06 and 1.29 in men and 1.19 and 1.62 in women, depending on the type of SLT product.^31^ This study did not report CIs or *P* values. Age-specific mortality rates are higher among male smokers than nonsmokers, but most concerning is that this difference is greater in younger age-groups (35-54 years).^31^ This premature mortality contributes to years of life lost and loss of productivity and impairs the ability to achieve Sustainable Development Goal (SDG) 3, which is to ensure healthy lives and promote well-being for all at all ages.^32^ The cancer mortality data are derived from PBCRs and include data from municipal corporations. These data are again an underestimate because death registration is often incomplete.^33^ Thus, unlike European and American countries, which have comprehensive death registration and provide good-quality data, these data lack precision because of incomplete death registration.^34^

#### Survival.

Approximately 60%-80% of OC cases in India are diagnosed at stages III and IV compared with 40% in developed countries.^7,35^ It is well documented that diagnosis at an advanced stage is an important cause of poor survival because many lesions are very aggressive and cause local invasion and metastasis.^36^ The 5-year survival rate for localized tongue cancers is 54.3% but is 3.1% for advanced stages.^33^ For localized disease at other sites in the oral cavity, the 5-year survival rate is 60.2% but is 3.3% for advanced stages.^33^ Tongue cancers are the predominant type of OCs and the main cancer site not only for OC but also for head and neck cancers.^37^ Tongue cancers are more likely to metastasize early probably because of the rich blood supply and lymphatic drainage of the tongue.^3^ Compared with other cancers with distant metastasis, OCs have the lowest survival rate.^33^ Within India, there are marked variations in survival between rural and urban areas, although the differences across metropolitan areas were small, and detailed data were not provided by the authors.^33^ Overall, many OCs are diagnosed in advanced stages because of poor access to medical care and/or delays in seeking medical care.

### Socioeconomic Burden

OC is associated with poverty.^38^ Both within and between countries, the prevalence and distribution of the risk factors parallel socioeconomic development.^17^ The greatest OC burden exists among the lower socioeconomic groups^7^ because they are generally more exposed to the risk factors^26^ and have limited access to prevention and treatment.

The government’s expenditure per patient with cancer in India is US $641, which is very low compared with the expenditure of other countries with a high cancer burden.^39^ In India, public health spending is 1.15% of gross domestic product (GDP), which is one of the lowest proportions in the world. Thus, most of the spending on health care in India is out of pocket. Health care costs involve not only medical expenses but also indirect costs, such as loss of productivity because of high rates of morbidity and mortality.^40^ This imposes a huge burden on the individual, family, society, and nation and exacerbates poverty. It is a vicious cycle: tobacco use by the poor, reduced capacity to spend on essentials, and exacerbation of poverty by tobacco-related illness. Even for wealthy countries with well-developed health care infrastructures and the ability to spend 6%-16% of GDP, cancer management is not a trivial problem.^23^ The magnitude of the problem in a low-resource setting with high exposure to risk factors coupled with social acceptability of risk factors, poverty, illiteracy, lack of awareness, and lack of affordability and accessibility to health care means that a need exists to focus on primary and secondary prevention.

### Opportunities

Oral carcinogenesis (squamous cell carcinoma) is a multistep process, with multifocal transformation of normal mucosa to a potentially malignant stage followed by carcinoma.^41^ Thus, OCs are preceded for varying periods by clinically detectable chronic lesions called premalignant lesions and conditions, currently grouped under the term OPMDs.^42^ Because OPMDs are often asymptomatic, medical help is not sought until persistent pain or functional disturbance develops.^43^ A 10-year follow-up study in India showed that all newly detected OC cases had developed from previously diagnosed precancerous lesions/conditions.^11^ A study from Ahmedabad reported an increased incidence of OC among young men as a result of increasing consumption of areca nut, with associated submucous fibrosis.^12^ Such studies emphasized the need for detection of precancer and early cancer. The natural history of OPMDs shows that malignant transformation may take from 5 to 10 years.^43,44^ The long delays in the malignant transformation of OPMDs provide an opportunity for prevention of OC through treatment of OPMDs.^45^ The presence of an OPMD is, itself, a risk factor for OC. Regression of OPMDs can potentially prevent OC.^46^ Identification and management of OPMDs is vital to OC control^41,45,47,48^ and is an important approach to secondary prevention of OC.^41,49^ India is reported to have a high incidence of OPMDs that progress to OC,^44^ so early detection of OPMDs is a potentially effective cancer control measure.^50^ Early detection and intervention at one or more steps of the multistep carcinogenesis can impede OC development from the putative OPMDs.^41^

### OC Screening

OC screening involves oral examination or application of a test for identifying changes in the oral mucosa that may precede or predict development of OC.^14^ Some countries have integrated oral screening into general health screening,^46,51^ although this is not widely available. OC etiology and epidemiology have received substantial attention among researchers, which has increased knowledge about the disease, its associated economic burden, and implications for Indian health care and society. It is critical to track OPMDs particularly because they fulfill many of the criteria for screening,^52^ including some characteristics of the disease, namely a preclinical phase that is usually undiagnosed but, if detected early, will result in a better outcome than later treatment and a simple and cost-effective screening test. As a national priority, high-level meetings have recommended screening programs.^53^ Although there are recommendations for a program of early detection, few studies of screening in India have been published; one randomized controlled trial showed no effect of screening on mortality.^54^ In spite of the magnitude of the disease burden compared with other cancers amenable to early detection, there are no guidelines^5^ to develop a framework for OC screening in India; this may be due to the fact that OC screening has not been shown to reduce mortality in the general population and does not fulfill all the criteria for population screening.^14^ Even from the first regional consultation of the International Cancer Screening Network in India, there were no reports on screening for OC,^55^ although an operational framework has been developed.^15^

Cuba is the only country to have conducted population-based OC screening, and although there was a significant reduction in advanced-stage cancers, no change in OC incidence and mortality could be ascribed to the screening program.^5^,^6^^,^^5^,^7^ A randomized controlled trial of OC screening was conducted in India, with OC mortality as the primary outcome; it reported a statistically significant reduction in male mortality among high-risk groups but did not show any statistically significant reduction in mortality overall.^54^ Even if it had produced a clear finding, the trial was inappropriately designed to provide evidence of efficacy for a national screening program.^14^ Nonetheless, on the basis of the inference that there was a reduction in mortality among high-risk patients with OC in this Indian trial, screening programs for high-risk groups were introduced in Taiwan^58^ and Sri Lanka.^46^

For more than 3 decades, WHO has continued to emphasize the role of dentists in OC control.^59^ Because OC control is necessarily multidisciplinary, dentists play a vital role, both independently and with the oral health team, in primary, secondary, and tertiary OC prevention as well in preventing post-therapeutic complications by providing prophylactic and continued oral care.^60,61^ Elaboration on the role of dentists in multiple levels of patient care is beyond the scope of this article and is described elsewhere.^62^ With an understanding of the importance of dentists and their role in early detection, high-income countries have strengthened and used their dental workforce in opportunistic OC screening. A study from the United Kingdom demonstrated the feasibility of opportunistic screening in dental practices as an alternative to a population-based screening program,^63^ and a review from Europe, which compared various screening models, recommended opportunistic screening in dental practices.^13^ Conventional oral examination has been proven to be the most efficient and cost-effective method for detecting OC,^64,65^ and this has been the approach in all studies. Studies from India have reported on the use of mobile technology (mHealth) and community health workers for OC screening^66,67^ as well as a role for dentists and dental schools in providing remote assistance in an mHealth OC screening project.^67,68^ A review from the deliberations of a workshop by the Indian Institute for Cytology and Preventive Oncology, in collaboration with the US National Cancer Institute Center for Global Health, on screening and early detection for common cancers in India recommended opportunistic screening of high-risk adults with the involvement of dentists using conventional oral examination.^5^

In India, there are no population registries of high-risk individuals; such a health information registry needs an organized, systematic health care system. With minimal health funding from the central government, widespread targeted screening may not be practical. Rather, opportunistic regional screening could be a better way to approach a screening program, particularly because lessons learned could be applied in a sequential fashion in any one region and subsequently transferred from region to region. Although there have been recommendations for early detection,^69^ OC screening initiatives have not been instituted.^70,71^

### Challenges in India

An organized health care system is a primary requirement for any screening program.^46^ Fragmented, low-resource settings that lack workforce and technical facilities—and that are all too common in developing countries—are barriers to implementing cancer screening programs.^46^ Developed countries are more likely to have the capacity to assess the population and establish who is at risk for particular diseases, including cancers.^72^ Many high-income countries have a well-developed health information system^46^ that can identify eligible individuals and allow screening at regular intervals. Low- and middle-income countries, such as India, do not have such health information systems, and there is geographically inequitable distribution of health care services, making health care accessibility uneven. The cultural diversity in India aggravates the complexities associated with risk factors, such as tobacco (smoked and smokeless) and areca nut. The widespread acceptance of SLT and areca nut poses a greater challenge for control of these risk factors. Because there are regional variations in SLT use, a need exists for regional control programs rather than for a single national system. Tobacco and areca nut are used in various combinations, and our current research program (unpublished results) also has identified combined use of areca nut and tobacco in sachets. Both are major risk factors for OC, and there is need to integrate tobacco and areca nut control strategies in the pursuit of effective OC control in India. Although tobacco control programs are widely available, there are no control programs for areca nut; however, the need for a WHO framework on areca nut has been identified.^73^ For India, there are greater challenges because of insufficient workforce and technical facilities for the required multipronged approach to OC control. Although WHO has recommended screening as part of cancer control programs and has urged member states to make this a national health priority,^26^ and although the government of India envisages free OC screening,^74^ the present 1.15% of GDP allocated to health care is likely to undermine the possibility of screening programs for the large and rapidly increasing burden of OC.

## DISCUSSION

As evidenced from the literature, OC is an established public health burden in India. The epidemic of OC reported 2 decades ago^75^ has burgeoned and continues to grow at an explosive rate among young Indians,^50,76,77^ and with an aging population, OC incidence is projected to increase.^27,78^ The projected numbers of OC cases will impose huge family, societal, and economic burdens that are insurmountable for a low-resource country. This is also a barrier to achieving SDG 1 (to end poverty in all its forms everywhere) and SDG 3 (to ensure healthy lives and promote well-being for all at all ages).^32,46,79,80^

Because OC is amenable to treatment when detected early or at the level of OPMDs, early detection is the key to effectively reducing the OC burden in India. Screening is an important cancer control strategy; thus, it is essential to determine the best possible pragmatic approach. There is a lack of evidence to support the implementation of a population-based screening program, and with the practical difficulties of undertaking another randomized controlled trial, an alternate method of cancer screening that deserves attention is the opportunistic screening of high-risk groups in a dental setting. The efficiency of dentists in OC screening is widely reported, and they play a vital role in OC control.^62^ After diagnosis, it is important to treat and maintain follow-up.^45^ There are recommendations for opportunistic oral precancer screening at dental settings in India.^5^ Studies from India have reported on pilot studies of telementoring health care providers^81^ and training frontline health care providers in using mHealth^68^ to facilitate opportunistic OC screening. Studies from the United Kingdom, Europe, and Japan have shown the feasibility of oral precancer screening by conventional oral examination as a practical and cost-effective method. Adaptation of these screening programs in India will be helpful; however, unlike some developed countries where systematic annual oral health programs and population databases allow invitation-based screening, in India, the best initial direction is an opportunistic screening program. Even with that approach, limited capacity for out-of-pocket health care spending may pose a barrier to the uptake of a voluntary program in a wider dental health care setting. Other barriers are wide variation in socioeconomic characteristics, health-seeking behavior, levels of health literacy, and accessibility of health care between the states.

Dental colleges located in various districts of most states in India could be an alternative location for opportunistic oral precancer screening programs. In this setting, regional variations in risk factors (tobacco [smoked and smokeless], areca nut, etc) could also be addressed, and thus, tailored cancer prevention strategies could be provided without additional consultation cost to the patient. Screen-detected lesions can also be treated onsite. For detected OCs, dental colleges are highly valuable in their ability to provide preradiotherapeutic prophylaxis and postradiation stability.^61^ Repetitive screening, as required for the success of OC control,^82^ can be done as follow-up. On the basis of the experience of undertaking this review, we note that there are gaps in knowledge. For example, who among high-risk groups seeks dental care, what technical facilities are available for early detection, and what services are available and used for precancer detection by Indian dental patients? There is a need for research to clarify these issues before recommending opportunistic oral precancer screening programs. The literature search did not identify any studies on opportunistic screening in dental settings reported from India, but we are now undertaking a pilot project to assess the feasibility of a regional opportunistic oral precancer screening program in dental colleges. The aim is to provide information on whether an opportunistic oral precancer screening program could be a key pragmatic approach to regional OC control in India.
